# The incidence of shoulder arthroplasty infection presents a substantial economic burden in the United States: a predictive model

**DOI:** 10.1016/j.jseint.2023.03.013

**Published:** 2023-04-11

**Authors:** Samuel Schick, Joseph Elphingstone, Sudarsan Murali, Karen Carter, William Davis, Gerald McGwin, Thomas Evely, Brent Ponce, Amit Momaya, Eugene Brabston

**Affiliations:** a Department of Orthopaedic Surgery, University of Alabama at Birmingham, Birmingham, AL, USA; b Department of Epidemiology, University of Alabama at Birmingham School of Public Health, Birmingham, AL, USA; c Hughston Clinic, Columbus, GA, USA

**Keywords:** Shoulder arthroplasty, Infection, Prosthetic joint infection, Anatomic, Reverse, Hemiarthroplasty, Charges

## Abstract

**Background:**

Periprosthetic joint infections (PJIs) are a major cause of morbidity after shoulder arthroplasty. Prior national database studies have estimated the trends of shoulder PJI up to 2012.^21^ Since 2012, the landscape of shoulder arthroplasty has changed drastically with the expanding popularity of reverse total shoulder arthroplasty. The dramatic growth in primary shoulder arthroplasties is likely paralleled with an increase of PJI case volume. The purpose of this study is to quantify the rise in shoulder PJIs and the economic stress they currently place on the American healthcare system as well as the toll they will incur over the coming decade.

**Methods:**

The Nationwide Inpatient Sample database was queried for primary and revision anatomic total shoulder arthroplasty, reverse total shoulder arthroplasty, and hemiarthroplasty from 2011-2018. Multivariate regression was used to predict cases and charges through the year 2030 adjusted to 2021 purchasing power parity.

**Results:**

From 2011 to 2018, PJI was found to be 1.1% shoulder arthroplasties, from 0.8% (2011) to 1.4% (2018). Anatomic total shoulder arthroplasty experienced the greatest proportion of infections at 2.0%, followed by hemiarthroplasty at 1.0% and reverse total shoulder arthroplasty at 0.3%. Total hospital charges grew 324%, from $44.8 million (2011) to $190.3 million (2018). Our regression model projects 176% growth in cases and 141% growth in annual charges by 2030.

**Conclusion:**

This study demonstrates the large economic burden that shoulder PJIs pose on the American healthcare system, which is predicted to reach nearly $500 million in charges annually by 2030. Understanding trends in procedure volume and hospital charges will be critical in evaluating strategies to reduce shoulder PJIs.

Believed to be the first shoulder arthroplasty, Péan documented replacing the proximal humerus of a patient suffering from tuberculoid arthritis with a rubber and platinum prosthesis in 1893.^23^ Since then, numerous variations of shoulder implants have been devised and implemented; however, Neer’s anatomic total shoulder arthroplasty (ATSA)^19^ and Grammont’s reverse total shoulder arthroplasty (RTSA)^11^ techniques survived and have become the mainstay of glenohumeral joint replacement. RTSAs gained Food and Drug Administration approval in 2004 and were within the same International Classification of Diseases (ICD) procedure code as ATSAs until October 1, 2010.^13^ Since the Food and Drug Administration approval, surgical indications for RTSA have expanded^32^ and have prompted an exponential growth in cases, surpassing annual ATSA procedure volumes and even outpacing the growth of total hip and knee replacements.^31^

With the increased incidence of shoulder arthroplasty, case volume periprosthetic joint infections (PJIs) have risen in parallel.^7^ Although the reported infection rate is low following shoulder arthroplasty (0.7%-4.0%),^4^^,^^6^^,^^21^^,^^22^^,^^25^^,^^28^^,^^29^ periprosthetic infections are a major source of morbidity, with 50%-78% of infected primary arthroplasties requiring further revision^22^ and 25%-29% of multirevision cases being linked to PJI.^10^^,^^26^ For patients and the healthcare system, this can be financially devastating, as the hospital cost for a two-stage reimplantation for an infected shoulder arthroplasty has been estimated to be roughly twice that of a primary replacement.^1^ Addressing PJIs is of the utmost importance, given the dramatic rise in total shoulder arthroplasties with RTSAs projected to quadruple to nearly 300,000 cases per year by 2025.^31^

To capture the national landscape of shoulder periprosthetic infections in the United States, this study used data from the National Inpatient Survey (NIS). Over an 8-year survey period (2011-2018), PJI procedure volumes and total hospital charges for ATSA, RTSA, and hemiarthroplasty (HA) were evaluated. Additionally, predictive modeling was used to estimate future economic and healthcare burdens PJIs will pose over the coming decade, extending to the year 2030.

## Methods

### Study design

Deidentified, publicly available data from the NIS was used. The NIS samples from states participating in the Healthcare Cost and Utilization Project, which covers more than 97% of the US population. The database approximates 20% of hospital discharges nation-wide but does not include rehabilitation or long-term acute care hospitals. In our study, NIS was used to estimate annual volume and hospital charges associated with shoulder PJIs.

### Data collection

ICD 9th revision (ICD-9) and 10th revision (ICD-10) procedure codes for primary and revision ATSA, RTSA, and shoulder HA were used to identify all shoulder arthroplasty procedures. Periprosthetic infection cases were queried using arthroplasty procedure codes that were cross-referenced with periprosthetic and bone infection diagnosis codes from 2011 to 2018 NIS samples. A full listing of codes can be found in the supplemental file. ICD-9 revision codes, 81.83 and 81.97 (and their ICD-10 conversion equivalents), were not used in our search due to their overlapping billing terminology with elbow prosthesis revision and sternoclavicular and acromioclavicular joint repair.

### Statistical analysis

National estimates were calculated in SAS (SAS Institute Inc., Cary, NC, USA) using discharge weights provided by the NIS. The 2011 dataset was chosen as the initial search year because the ICD-9 procedure code (81.88) for RTSA was not distinct from the ATSA (81.80) until October 1, 2010.^18^ ICD-10 procedure and diagnosis codes were used to query for procedures occurring after the transition from ICD-9 on October 1, 2015.

Procedural volume, infection rate, and hospital charges were obtained for each prosthetic type PJI. The 95% confidence intervals (CIs) for annual total charges and procedure volumes were generated using NIS Charge and Discharge Weights. All charges were adjusted for January 2021 purchasing power parity using the Consumer Price Index for Medical Care to account for year-to-year inflation.^3^ Independent linear regression models were used to predict future volume and charges for each type of PJI to 2030.

## Results

**Figure 1:**
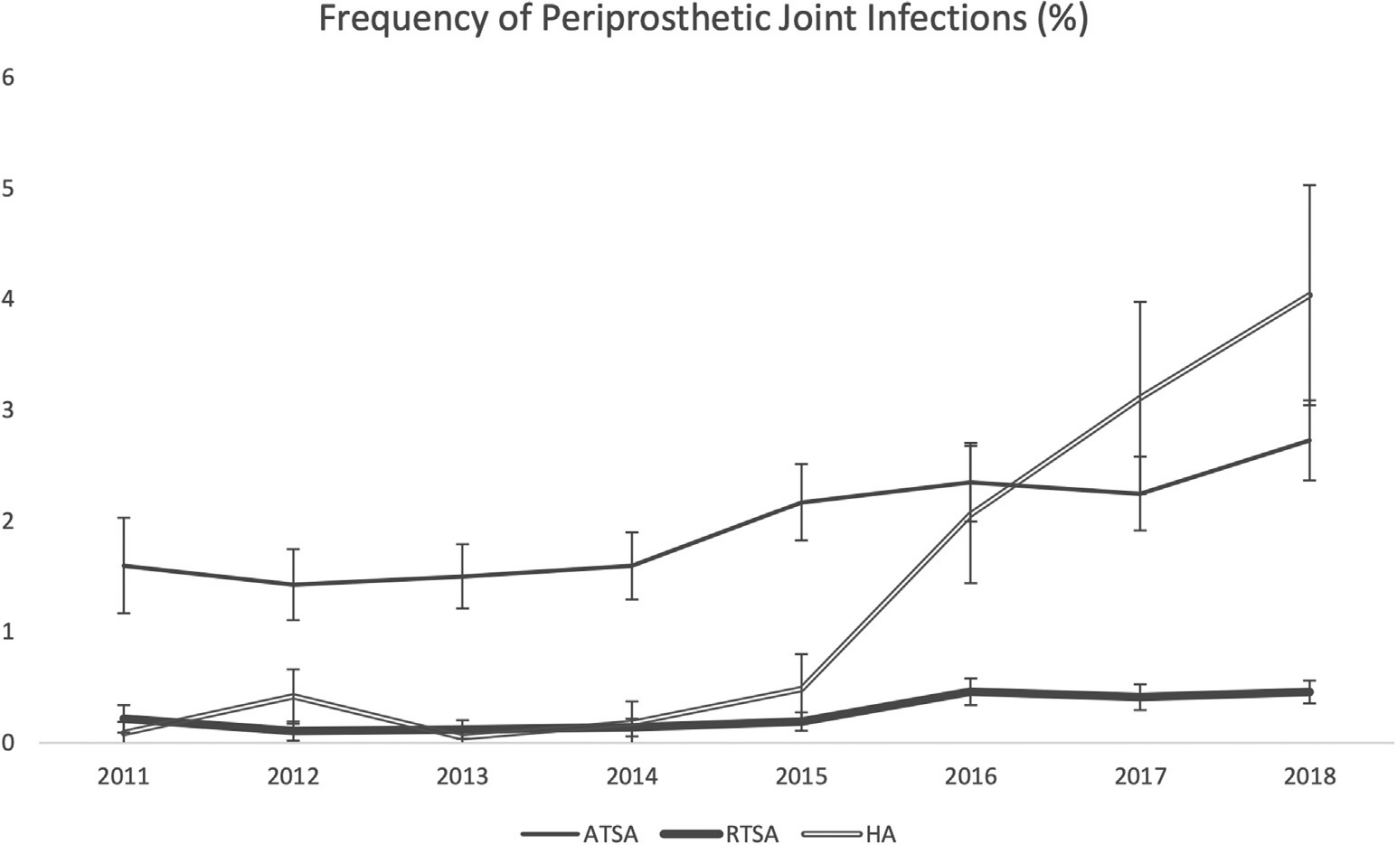
Periprosthetic joint infection rate from 2011 to 2018. *ATSA*, anatomic total shoulder arthroplasty; *RTSA*, reverse total shoulder arthroplasty; *HA*, hemiarthroplasty.

Over the 8-year sample, the total annual shoulder arthroplasty cases rose from 66,961 in 2011 to 127,440 in 2018, totaling 727,550 (95% CI 680,981-774,119) cases. Of these, there were 7733 (95% CI: 5968-9498) PJIs, indicating an average infection rate of 1.1% (0.8%-1.4%). From 2011 to 2014, annual infections were stable, slowly rising from 538 (0.8%) to 625 (0.8%) cases. However, annual PJIs rose to 930 (1.0%) in 2015 and continued growing to 1755 (1.4%) cases in 2018. When inspecting each prosthesis type PJI, ATSAs constituted the majority of PJI cases (74.8% of PJIs) and represented a 2.0% (95% CI 1.5%-2.5%) infection rate. During the sample period, annual ATSA PJI cases grew from 477 in 2011 to 1125 in 2018, representing an infection rate growth from 1.6% to 2.7%. RTSA PJI cases grew considerably but maintained a low infection rate, rising from 47 (0.2%) in 2011 to 365 (0.46%) in 2018. Similar to RTSA, HA PJI cases grew rapidly, from 14 (0.1%) in 2011 to 265 (4.0%) in 2018. However, unlike RTSAs, which experienced a 1600% (4719 to 79630) case growth, primary HAs declined by more than 60% (18,222 to 6565) over this period (Table I and Fig. 1).

**Table I:** Infected shoulder arthroplasty national procedure volume and charges (2021 USD).

Year	ATSA cases (95% CI)	Hospital charges ATSA (2021 USD) (95% CI) In millions	Infected RTSA Cases (95% CI)	Mean hospital charge of infected RTSA (95% CI) In millions	Infected HA Cases (95% CI)	Mean hospital charge of infected HA (95% CI) In millions
2011	477 ( 333-621)	39.5 (25.7-53.2)	47 (16-78)	4.3 (1.0-7.6)	14 (0-30)	1.1 (0-2.5)
2012	430 (327-534)	35.4 (25.2-45.5)	25 (3-47)	3.1 (0.3-5.9)	50 (19-81)	4.7 (1.5-7.9)
2013	500 (396-604)	38.3 (29.4-47.2)	35 (9-61)	3.8 (3.6-4.1)	5 (−5 to 15)	0.8 (−0.8 to 2.4)
2014	560 (446-674)	52.3 (37.8-66.9)	50 (19-81)	5.7 (1.7-9.6)	15 (−2 to 32)	1.2 (−0.3 to 2.7)
2015	800 (663-937)	92.4 (71.2-113.5)	90 (49-131)	10.8 (4.8-16.7)	40 (12-68)	4.9 (0.7-9.1)
2016	935 (783-1087)	118.3 (95.1-141.6)	265 (192-338)	46.0 (31.3-60.6)	175 (118-232)	24.4 (15.3-33.6)
2017	955 (802-1108)	88.3 (69.0-107.6)	275 (193-357)	31.3 (20.3-42.2)	235 (163-307)	25.4 (14.8-36.1)
2018	1125 (964-1286)	117.3 (94.7-139.8)	365 (278-452)	45.2 (32.0-58.5)	265 (192-338)	27.8 (18.3-37.3)
Change from 2011	+135.8%	+197.1%	+676.6%	+953.3%	+1792.9%	+2484.6%

*USD*, United States dollars; *ATSA*, anatomic total shoulder arthroplasty; *CI*, confidence interval; *RTSA*, reverse total shoulder arthroplasty; *HA*, hemiarthroplasty.

After adjusting for 2021 relative purchasing power parity,^3^ the average total hospital charge per PJI case was $106,311 (95% CI 76,572-136,050). When subdivided, ATSA, RTSA, and HA PJIs averaged total charges of $100,591 (95% CI: $77,485-$123,698), $130,290 ($82,421-$178,159), and $113,128 ($61,532-$164,723), respectively. From 2011 to 2018, charges per case increased considerably, with ATSAs rising 26% ($82,749 to $104,224), RTSAs by 35.6% ($91,362 to $123,916), and HA by 36.5% ($76,930 to $105,043). Although inpatient length of stay differed significantly between ATSA (4.7+/−10.9 days), RTSA (2.9+/−5.5 days), and HA (4.8+/−8.1) (*P* < .001), these differences were not enough to explain the evolution in charges over the 8-year sample period.

Nationally, the annual charges for all PJIs rose by more than 300% during the sample period, from $44.9 million (95% CI: $26.7-63.3 million) in 2011 to $190.3 million ($145.1-235.6 million) in 2018. This growth is attributed to a 2-fold increase in ATSA ($39.5 to $117.3 million), 10-fold growth of RTSA ($4.3 to $45.2 million), and 25-fold growth of HA ($1.1 to $27.8 million) total charges (Table I).

**Figure 2:**
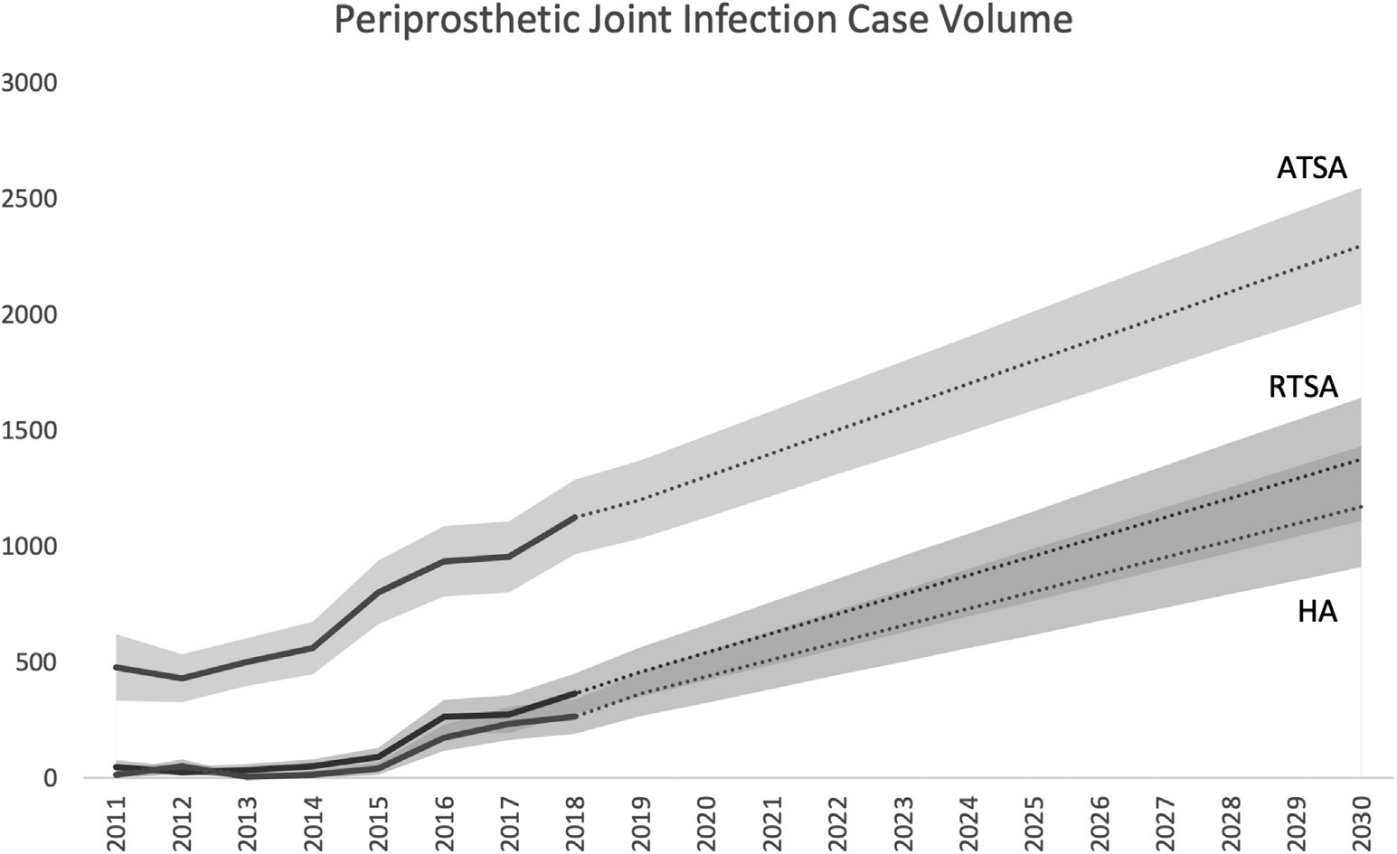
Periprosthetic joint infection case volume from 2011 to 2030. *ATSA*, anatomic total shoulder arthroplasty; *RTSA*, reverse total shoulder arthroplasty; *HA*, hemiarthroplasty.

Linear regression modeling projected the expected annual case volume and hospital charges for shoulder arthroplasty PJIs and indicated that by 2030, PJIs are expected to grow an additional 176% (4844 cases 95% CI 4067-5621). Individually, ATSA, RTSA, and HA PJI cases are expected to rise by 104% (to 2297 [CI 2047-2547]), 277% (1376 [1110-1642]), and 342% (1171 [910-1432]), respectively (Table II, Fig. 2).

**Table II:** Projected infected shoulder arthroplasty national procedure volume and charges (2021 USD).

Year	Infected ATSA cases (95% CI)	Hospital charges for infected ATSA (95% CI)	Infected RTSA Cases (95% CI)	Hospital charges for infected RTSA (95% CI)	Infected HA Cases (95% CI)	Hospital charges for infected HA (95% CI)
2019	1203 (1033-1372)	131.0 million (105.9-156.2)	458 (351-564)	47.5 million (32.3-62.6)	363 (267-458)	30.9 million (19.3-42.4)
2020	1302 (1125-1372)	144.0 million (117.0-171.0)	541 (420-662)	52.9 million (36.0-69.8)	436 (326-546)	35.2 million (22.3-48.2)
2021	1402 (1218-1585)	157.0 million (128.1-185.8)	625 (489-760)	58.3 million (39.6-77.0)	510 (384-635)	39.6 million (25.2-53.9)
2022	1501 (1310- 1692)	169.9 million (139.2-200.7)	708 (558-858)	63.8 million (43.3-84.2)	583 (442-724)	43.9 million (28.1-59.7)
2023	1601 (1402-1799)	182.9 million (150.3-215.5)	792 (627-956)	69.2 million (46.9-91.4)	657 (501-812)	48.3 million (31.1-65.5)
2024	1700 (1494-1906)	195.9 million (161.4-230.3)	875 (696-1054)	74.6 million (50.6-98.6)	730 (559-901)	52.6 million (34.0-71.2)
2025	1800 (1586-2013)	208.8 million (172.5-245.2)	959 (765-1152)	80.0 million (54.3-105.8)	804 (618-989)	57.0 million (37.0-77.0)
2026	1899 (1679-2120)	221.8 million (183.6-260.0)	1042 (834-1250)	85.5 million (57.9-113.1)	877 (676-1078)	61.3 million (39.9-82.8)
2027	1999 (1771-2226)	234.8 million (194.7-274.9)	1126 (903-1347)	90.9 million (61.6-120.2)	951 (735-1166)	65.7 million (42.8-88.5)
2028	2098 (1863-2333)	247.7 million (205.8-289.7)	1209 (972-1446)	96.3 million (65.2-127.4)	1024 (793-1255)	70.0 million (45.8-94.3)
2029	2198 (1955-2440)	260.7 million (216.9-304.5)	1293 (1041-1544)	101.7 million (68.9-134.6)	1098 (851-1343)	74.4 million (48.7-100.0)
2030	2297 (2047-2547)	273.7 million (228.0-319.4)	1376 (1110-1642)	107.2 million (72.5-141.8)	1171 (910-1432)	78.7 million (51.6-105.8)
Change from 2018	+104.1%	+133.4%	+277.0%	+136.9%	+341.9%	+182.8%

*USD*, United States dollars; *ATSA*, anatomic total shoulder arthroplasty; *CI*, confidence interval; *RTSA*, reverse total shoulder arthroplasty; *HA*, hemiarthroplasty.

**Figure 3:**
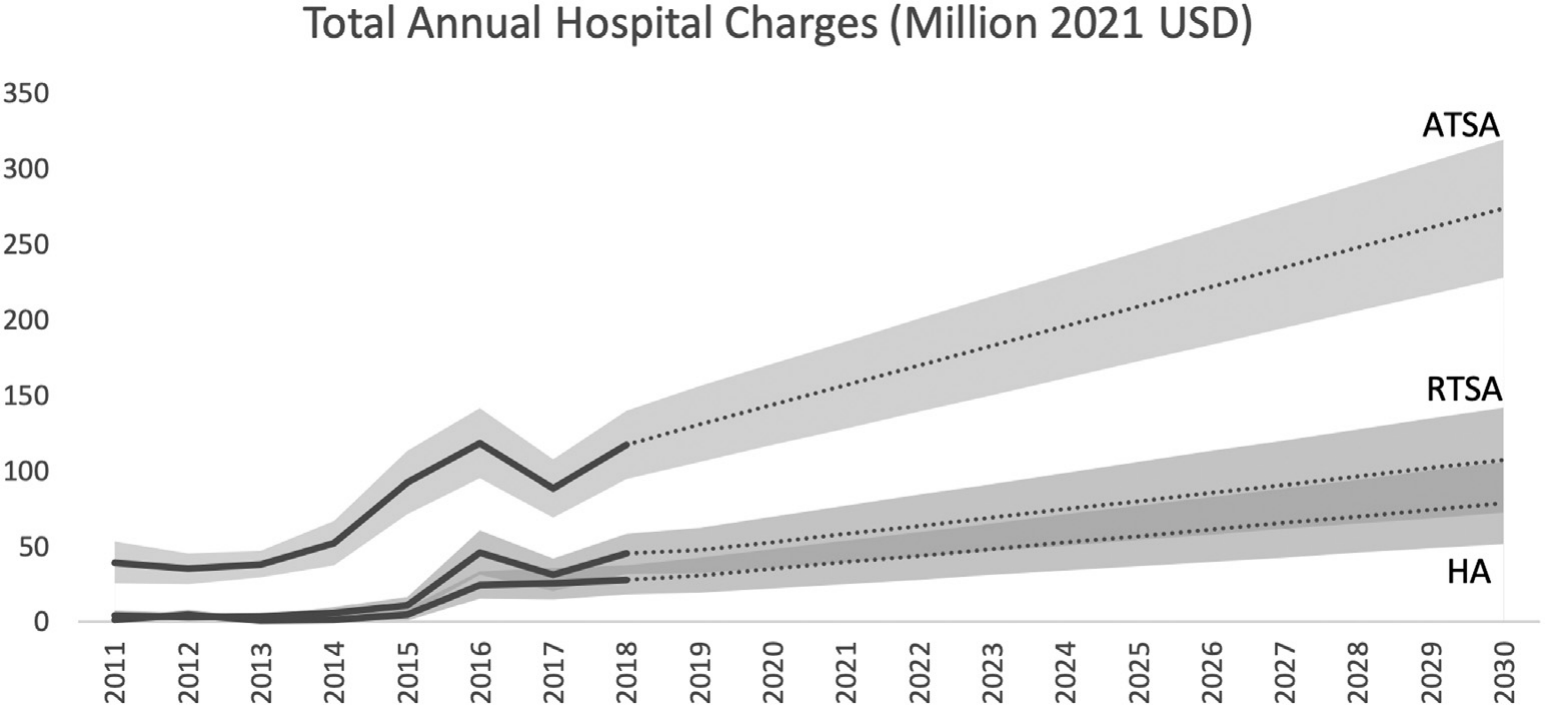
Total annual periprosthetic infection hospital charges (million USD) from 2011 to 2030. *ATSA*, anatomic total shoulder arthroplasty; *RTSA*, reverse total shoulder arthroplasty; *HA*, hemiarthroplasty; *USD*, United States dollars.

Projected inpatient charges for all infected shoulder arthroplasties are expected to rise by 141% to $459.6 million (95% CI: $352.1-$567.0 million) by 2030. Of this total, ATSAs are predicted to contribute $273.7 million ($228.0-$319.4 million), RTSAs $107.2 million ($72.5-$141.8 million), and HAs $78.7 million ($51.6-$105.8 million) (Table II, Fig. 3).

## Discussion

To our knowledge, this is the first study to estimate the current and future economic and healthcare burdens that shoulder PJIs place pose on the American healthcare system. Data from the NIS show that over the past decade, annual PJI cases have tripled, incurring more than $190 million in hospital charges in 2018. More so, PJIs are outpacing primary procedure growth, rising annually from a 0.8% rate in 2010 to 1.4% in 2018. Our data indicate that annual procedure volume and charges will expand to nearly $500 million in annual charges by 2030.

Our finding of a 1.1% shoulder PJI rate is consistent with previous estimates of 0.7% to 4.0%.^4^^,^^6^^,^^21^^,^^22^^,^^25^^,^^28^ In contrast to earlier reports, which suggest no difference in infection rates between arthroplasty subtypes,^8^^,^^22^^,^^30^ our results suggest differences in infection rates between ATSA, RTSA, and HA. Population differences in the Parada et al dataset, which includes patients from Europe, may explain these discordant findings. Additionally, indications for shoulder arthroplasty have expanded considerably in the past 2 decades in the United States. Different indications for surgical management of shoulder conditions may also play a role in the dissimilarities of infection rates by prothesis type seen in the United States.

Padegimas et al previously investigated shoulder PJI trends using the NIS database, sampling from 2002 to 2011.^21^ The group also demonstrated rising infection case volumes with a stable infection rate of 1.0%. With updated figures, our results indicate an evolution in this rate, growing from 0.8% in 2011 to 1.4% in 2018. This rise is likely due to recent surgical trends including an increase in complex pathology and expanding surgical indications. First is the distinction of RTSA procedure coding. Since the distinction of an RTSA ICD-9 code (81.88) in the fourth quarter of 2010,^18^ and the increased surgical indications for RTSAs, the procedure has experienced a meteoric rise in cases, increasing by 191.3% from 2011 to 2017.^31^ In addition to managing rotator cuff arthropathy and revision arthroplasty, RTSAs have been used increasingly for comminuted proximal humerus fractures, complex bone loss, and revision shoulder arthroplasty.^32^ In line with Dillon et al’s report that RTSAs surpassed HAs for proximal humerus fractures reconstruction for the first time in 2015,^5^ we noted a tripling of infected RTSA cases during this time period. This finding may coincide with the reports of several small patient cohorts which have demonstrated infection rates of 6.25% to 11.0% following RTSA for proximal humerus fractures.^2^^,^^9^^,^^17^ Additionally, the changes in cases may also correspond with a switch to more precise coding from ICD-9 to ICD-10 codes in the fourth quarter of 2015.

We noted a dramatic rise in the HA infection case volume, with primary procedures declining over the past decade as infection procedures grew. However, in light of previous work indicating a stable 20-year infection rate of 1.3% follow-up for HA,^28^ we believe that the expansion in PJI cases may be reflecting the growing use of HA for revision in those with complex glenoid morphology rather than a precipitously rise in infected primary procedures. The precipitous rise of HA procedures may be a result of antibiotic spacers coded as HA, and this may represent a much larger rise in infection than our model estimates. Although some view RTSAs as the reimplant of choice, HAs are a reasonable option for joint salvage for those with substantial glenoid bone loss.^12^^,^^15^^,^^16^ However, if the issue of glenoid bone erosion is not addressed with a prosthesis, bone loss is likely to worsen.^15^ This approach has demonstrated less short-term erosion compared to HA without glenoid arthroplasty.^12^ Given the indications for HA, it appears that surgeons may be reserving HA as a worst case scenario for revision.

Although Padegimas et al evaluated PJI average procedure costs, and charges,^21^ to our knowledge, the present study is the first to estimate the national economic burden of shoulder PJI in the United States. With the rise in inflation of the USD in 2021, the average adjusted charge per case for all shoulder PJI was substantially more in 2018 ($106,311) than Padegimas reported in 2015 with 2011 data ($42,249).^21^ In the present study, we indicate that nation-wide hospital charges for PJIs have increased by 300% over the 8-year sample. Unfortunately, this burden is expected to continue growing, with charges projected to approach $500 million dollars in 2030. Worse yet, the charges outlined by the NIS database underestimate the true total cost, as they do not account for physical therapy, additional clinic visits, and loss of time at work. Hip and knee literature reiterates this burden, with combined total knee and hip PJI charges expected to rise by 86% to nearly $1.9 billion dollars in 2030.^14^^,^^24^

Due to the growing burden that PJIs are placing on the US health system, knowing potential patient-associated risk factors could assist in targeting at-risk populations. Several studies have indicated that younger patients, males, longer operative times, and a history of nutritional deficiency, drug abuse, anemia, complicated diabetes mellitus, congestive heart failure, metabolic syndrome, or obesity are all risk factors for shoulder PJI.^20^^,^^21^ Additionally, microbial cultures positive for *Cutibacterium acnes* were found to be an independent risk factor for failed treatment of PJI.^20^ Preoperative reduction in host colonization through topical benzoyl peroxide has been shown to effectively reduce shoulder PJI.^27^ Risk reduction should also include optimization of other modifiable patient factors and shared decision-making.

This study is not without limitations. Although the NIS is one of the largest national surgical databases available, it lacks the ability to distinguish replacement of a currently infected ATSA/RTSA/HA prosthesis and the replacement of an infected prosthesis with an ATSA/RTSA/HA prosthesis. Additionally, there is a possibility that HA is capturing cases from surgeons coding antibiotic spacer placement as HA. As shoulder arthroplasty is increasingly transitioning to an outpatient procedure, these infection rate and cost estimates may underestimate the true rates seen in outpatient surgery centers. Within the charge data are the institutional costs for hospitalization, but do not account for physician services, including that of the surgeon, anesthesiologist, infectious disease physician, and other medical specialists or additional outpatient medical services (physical therapy, follow-up medical appointments, and home healthcare) following hospital discharge. The NIS database also does not distinguish more than 1 PJI-related procedure for the same patient, thus will underpredict subsequent operations, the indication for the prosthesis, or the infectious etiology, all of which are important for better understanding of the epidemiology of shoulder PJIs. Finally, the projections included fixed rates of inflation and may not account for unforeseen macroeconomic events or changes in consumer habits and purchasing power.

## Conclusion

In summary, our study reveals the significant and growing strain that periprosthetic shoulder infections place on the American healthcare system, incurring $190 million in 2018 with projections estimating $500 million in hospital charges by 2030. We also indicate that PJIs have been outpacing primary procedures each year, growing from 0.8% of cases in 2011 to 1.4% in 2018. In light of the projected burden, healthcare system changes are needed to lessen the future burden placed by shoulder PJIs.

## Disclaimers

Funding: No funding was disclosed by the authors.

Conflicts of interest: The authors, their immediate families, and any research foundation with which they are affiliated have not received any financial payments or other benefits from any commercial entity related to the subject of this article.
