# Supplementary material for: The incidence of shoulder arthroplasty infection presents a substantial economic burden in the United States: a predictive model
Source: JSES Int. 2023 Apr 11;7(4):636–41. doi: 10.1016/j.jseint.2023.03.013 (PMC10328787; doi:10.1016/j.jseint.2023.03.013)
Supplement: Supplementary Table 1 [file mmc1.docx]

| **Group** | **ICD-9  (2002-2015 Q3)** | **ICD-10 (2015 Q4-2018)** |
| --- | --- | --- |
| 1.  **Anatomic Total Shoulder Arthroplasty (aTSA)** | **PX**  81.80 – other total shoulder arthroplasty  WITHOUT **DX**  996.66 - Infection and inflammatory reaction due to internal joint prosthesis  73001 - Acute osteomyelitis  73011 - Chronic osteomyelitis  73021 - Unspecified osteomyelitis  73091 - Unspecified infection of bone  73081 - Other infections involving bone in diseases classified elsewhere, shoulder region  99851 - Infected postoperative seroma | **PX**  0RRJ0JZ - Replacement of Right Shoulder Joint with Synthetic Substitute, Open Approach  0RRK0JZ - Replacement of Left Shoulder Joint with Synthetic Substitute, Open Approach  WITHOUT  **DX**  T84.50XA - Infection and inflammatory reaction due to unspecified internal joint prosthesis, initial encounter  T84.59XA - Infection and inflammatory reaction due to other internal joint prosthesis, initial encounter  M86119 - Other acute osteomyelitis, unspecified shoulder.  M86219 - Subacute osteomyelitis, unspecified shoulder.  M86619 - Other chronic osteomyelitis, unspecified shoulder  T81.4XXA -  Infection following a procedure, initial encounter |
| 2.  **Reverse Total Shoulder Arthroplasty (rTSA)** | **PX**  81.88 - Reverse total shoulder replacement  WITHOUT **DX**  996.66 - Infection and inflammatory reaction due to internal joint prosthesis  73001 - Acute osteomyelitis  73011 - Chronic osteomyelitis  73021 - Unspecified osteomyelitis  73091 - Unspecified infection of bone  73081 - Other infections involving bone in diseases classified elsewhere, shoulder region  99851 - Infected postoperative seroma | **PX**  0RRK00Z - Replacement of Left Shoulder Joint with Reverse Ball and Socket Synthetic Substitute, Open Approach  0RRJ00Z - Replacement of Right Shoulder Joint with Reverse Ball and Socket Synthetic Substitute, Open Approach  WITHOUT  **DX**  T84.50XA - Infection and inflammatory reaction due to unspecified internal joint prosthesis, initial encounter  T84.59XA - Infection and inflammatory reaction due to other internal joint prosthesis, initial encounter  M86119 - Other acute osteomyelitis, unspecified shoulder.  M86219 - Subacute osteomyelitis, unspecified shoulder.  M86619 - Other chronic osteomyelitis, unspecified shoulder  T81.4XXA -  Infection following a procedure, initial encounter |
| 3.  **Hemiarthroplasty**  **(HA)** | **PX**  81.81 - Arthroplasty; Partial shoulder replacement  WITHOUT **DX**  996.66 - Infection and inflammatory reaction due to internal joint prosthesis  73001 - Acute osteomyelitis  73011 - Chronic osteomyelitis  73021 - Unspecified osteomyelitis  73091 - Unspecified infection of bone  73081 - Other infections involving bone in diseases classified elsewhere, shoulder region  99851 - Infected postoperative seroma | **PX**  0RRK0J6 - Replacement of Left Shoulder Joint with Synthetic Substitute, Humeral Surface, Open Approach  0RRJ0J6 - Replacement of Right Shoulder Joint with Synthetic Substitute, Humeral Surface, Open Approach.  0RRK0J7 - Replacement of Left Shoulder Joint with Synthetic Substitute, Glenoid Surface, Open Approach  0RRJ0J7 - Replacement of Right Shoulder Joint with Synthetic Substitute, Glenoid Surface, Open Approach  WITHOUT **DX**  T84.50XA - Infection and inflammatory reaction due to unspecified internal joint prosthesis, initial encounter  T84.59XA - Infection and inflammatory reaction due to other internal joint prosthesis, initial encounter  M86119 - Other acute osteomyelitis, unspecified shoulder.  M86219 - Subacute osteomyelitis, unspecified shoulder.  M86619 - Other chronic osteomyelitis, unspecified shoulder  T81.4XXA -  Infection following a procedure, initial encounter |
| 4.  **Infected aTSA** | **DX**  996.66 - Infection and inflammatory reaction due to internal joint prosthesis  73001 - Acute osteomyelitis  73011 - Chronic osteomyelitis  73021 - Unspecified osteomyelitis  73091 - Unspecified infection of bone  73081 - Other infections involving bone in diseases classified elsewhere, shoulder region  99851 - Infected postoperative seroma  **AND PX**  **81.80 -** Arthroplasty; other total shoulder replacement  **80.01 -** arthrotomy for removal of prosthesis without replacement, shoulder | **DX**  T84.50XA - Infection and inflammatory reaction due to unspecified internal joint prosthesis, initial encounter  T84.59XA - Infection and inflammatory reaction due to other internal joint prosthesis, initial encounter  M86119 - Other acute osteomyelitis, unspecified shoulder.  M86219 - Subacute osteomyelitis, unspecified shoulder.  M86619 - Other chronic osteomyelitis, unspecified shoulder  T81.4XXA -  Infection following a procedure, initial encounter  **AND PX**  0RRJ0JZ - Replacement of Right Shoulder Joint with Synthetic Substitute, Open Approach  0RRK0JZ - Replacement of Left Shoulder Joint with Synthetic Substitute, Open Approach  [0RPJ0JZ](https://www.icd10data.com/ICD10PCS/Codes/0/R/P/J/0RPJ0JZ) - Removal of Synthetic Substitute from Right Shoulder Joint, Open Approach  [0RPK0JZ](https://www.icd10data.com/ICD10PCS/Codes/0/R/P/K/0RPK0JZ) - Removal of Synthetic Substitute from Left Shoulder Joint, Open Approach  0RPJ3JZ - Removal of Synthetic Substitute from Right Shoulder Joint, Percutaneous Approach  0RPJ4JZ - Removal of Synthetic Substitute from Right Shoulder Joint, Percutaneous Endoscopic Approach  0RPK3JZ - Removal of Synthetic Substitute from Left Shoulder Joint, Percutaneous Approach  0RPK4JZ - Removal of Synthetic Substitute from Left Shoulder Joint, Percutaneous Endoscopic Approach |
| 5.  **Infected rTSA** | **DX**  996.66 - Infection and inflammatory reaction due to internal joint prosthesis  73001 - Acute osteomyelitis  73011 - Chronic osteomyelitis  73021 - Unspecified osteomyelitis 73091 - Unspecified infection of bone  73081 - Other infections involving bone in diseases classified elsewhere, shoulder region  99851 - Infected postoperative seroma  **AND PX**  **81.80 -** Arthroplasty; other total shoulder replacement  **80.01 - A**rthrotomy for removal of prosthesis without replacement, shoulder | **DX**  T84.50XA - Infection and inflammatory reaction due to unspecified internal joint prosthesis, initial encounter  T84.59XA - Infection and inflammatory reaction due to other internal joint prosthesis, initial encounter  M86119 - Other acute osteomyelitis, unspecified shoulder.  M86219 - Subacute osteomyelitis, unspecified shoulder.  M86619 - Other chronic osteomyelitis, unspecified shoulder  T81.4XXA -  Infection following a procedure, initial encounter  **AND PX**  0RRK00Z - Replacement of Left Shoulder Joint with Reverse Ball and Socket Synthetic Substitute, Open Approach  0RRJ00Z - Replacement of Right Shoulder Joint with Reverse Ball and Socket Synthetic Substitute, Open Approach  [0RPJ0JZ](https://www.icd10data.com/ICD10PCS/Codes/0/R/P/J/0RPJ0JZ) - Removal of Synthetic Substitute from Right Shoulder Joint, Open Approach.  0RPJ3JZ - Removal of Synthetic Substitute from Right Shoulder Joint, Percutaneous Approach  0RPJ4JZ - Removal of Synthetic Substitute from Right Shoulder Joint, Percutaneous Endoscopic Approach  0RPK3JZ - Removal of Synthetic Substitute from Left Shoulder Joint, Percutaneous Approach  0RPK4JZ - Removal of Nonautologous Tissue Substitute from Left Shoulder Joint, Percutaneous Endoscopic Approach |
| 6.  **Infected**  **HA** | **DX**  996.66 - Infection and inflammatory reaction due to internal joint prosthesis  73001 - Acute osteomyelitis  73011 - Chronic osteomyelitis  73021 - Unspecified osteomyelitis 73091 - Unspecified infection of bone  73081 - Other infections involving bone in diseases classified elsewhere, shoulder region  99851 - Infected postoperative seroma  **AND**  **81.80 -** Arthroplasty; other total shoulder replacement  **80.01 -** arthrotomy for removal of prosthesis without replacement, shoulder | **DX**  T84.50XA - Infection and inflammatory reaction due to unspecified internal joint prosthesis, initial encounter  T84.59XA - Infection and inflammatory reaction due to other internal joint prosthesis, initial encounter  M86119 - Other acute osteomyelitis, unspecified shoulder.  M86219 - Subacute osteomyelitis, unspecified shoulder.  M86619 - Other chronic osteomyelitis, unspecified shoulder  T81.4XXA -  Infection following a procedure, initial encounter  **AND PX**  0RRK0J6 - Replacement of Left Shoulder Joint with Synthetic Substitute, Humeral Surface, Open Approach  0RRJ0J6 - Replacement of Right Shoulder Joint with Synthetic Substitute, Humeral Surface, Open Approach  0RRK0J7 - Replacement of Left Shoulder Joint with Synthetic Substitute, Glenoid Surface, Open Approach  0RRJ0J7 - Replacement of Right Shoulder Joint with Synthetic Substitute, Glenoid Surface, Open Approach  [0RPJ0JZ](https://www.icd10data.com/ICD10PCS/Codes/0/R/P/J/0RPJ0JZ) - Removal of Synthetic Substitute from Right Shoulder Joint, Open Approach.  [0RPK0JZ](https://www.icd10data.com/ICD10PCS/Codes/0/R/P/K/0RPK0JZ) - Removal of Synthetic Substitute from Left Shoulder Joint, Open Approach  0RPJ3JZ - Removal of Synthetic Substitute from Right Shoulder Joint, Percutaneous Approach  0RPJ4JZ - Removal of Synthetic Substitute from Right Shoulder Joint, Percutaneous Endoscopic Approach  0RPK3JZ - Removal of Synthetic Substitute from Left Shoulder Joint, Percutaneous Approach  0RPK4JZ - Removal of Synthetic Substitute from Left Shoulder Joint, Percutaneous Endoscopic Approach |
| 7.  **Infected**  **Revision Shoulder Arthroplasty** | **DX**  V43.61- Shoulder joint replacement  996.66 - Infection and inflammatory reaction due to internal joint prosthesis  73001 - Acute osteomyelitis  73011 - Chronic osteomyelitis  73021 - Unspecified osteomyelitis  73091 - Unspecified infection of bone  73081 - Other infections involving bone in diseases classified elsewhere, shoulder region  99851 - Infected postoperative seroma  AND **PX**  81.97 - Revision of joint replacement of upper extremity  80.01 - arthrotomy for removal of prosthesis without replacement, shoulder | **DX**  T84.50XA - Infection and inflammatory reaction due to unspecified internal joint prosthesis, initial encounter  T84.59XA - Infection and inflammatory reaction due to other internal joint prosthesis, initial encounter  M86119 - Other acute osteomyelitis, unspecified shoulder.  M86219 - Subacute osteomyelitis, unspecified shoulder.  M86619 - Other chronic osteomyelitis, unspecified shoulder  T81.4XXA -  Infection following a procedure, initial encounter  AND**PX**  0RWJ0JZ - Revision of Synthetic Substitute in Right Shoulder Joint, Open Approach  0RWK0JZ - Revision of Synthetic Substitute in Left Shoulder Joint, Open Approach  0RPJ0JZ - Removal of Synthetic Substitute from Right Shoulder Joint, Open Approach  0RPK0JZ - Removal of Synthetic Substitute from Left Shoulder Joint, Open Approach  0RPJ3JZ - Removal of Synthetic Substitute from Right Shoulder Joint, Percutaneous Approach  0RPJ4JZ - Removal of Synthetic Substitute from Right Shoulder Joint, Percutaneous Endoscopic Approach  0RPK3JZ - Removal of Synthetic Substitute from Left Shoulder Joint, Percutaneous Approach  0RPK4JZ - Removal of Synthetic Substitute from Left Shoulder Joint, Percutaneous Endoscopic Approach  0RWK3JZ - Revision of Synthetic Substitute in Left Shoulder Joint, Percutaneous Approach  0RWJ4JZ - Revision of Synthetic Substitute in Right Shoulder Joint, Percutaneous Endoscopic Approach |
